# D-2-Hydroxyglutarate producing neo-enzymatic activity inversely correlates with frequency of the type of isocitrate dehydrogenase 1 mutations found in glioma

**DOI:** 10.1186/2051-5960-2-19

**Published:** 2014-02-14

**Authors:** Stefan Pusch, Leonille Schweizer, Ann-Christin Beck, Johanna-Marie Lehmler, Susanne Weissert, Jörg Balss, Aubry K Miller, Andreas von Deimling

**Affiliations:** 1 German Consortium of Translational Cancer Research (DKTK), Clinical Cooperation Unit Neuropathology, German Cancer Research Center (DKFZ), INF 280, Heidelberg D-69120, Germany; 2 Department of Neuropathology, Institute of Pathology, INF 224, Ruprecht-Karls-University Heidelberg, Heidelberg D-69120, Germany; 3 Cancer Drug Development, German Cancer Research Center (DKFZ), INF 580, Heidelberg D-69120, Germany

**Keywords:** IDH1, 2-HG, D-2-hydroxyglutarate, Enzymatic activity, K_M_, Astrocytoma, Oligodendroglioma

## Abstract

**Background:**

IDH mutations frequently occur in diffuse gliomas and result in a neo-enzymatic activity that results in reduction of α-ketoglutarate to D-2-hydroxyglutarate. In gliomas, the frequency of IDH1 mutations in codon 132 increases in the order R132L-R132S-R132G-R132C-R132H with R132H constituting more than 90% of all IDH1 mutations.

**Results:**

We determined the levels of D-2-hydroxyglutarate in glioma tissues with IDH1 mutations. D-2-hydroxyglutarate levels increased in the order of R132H-R132C-R132S/R132G/R132L. We expressed and purified IDH1 wild type and mutant protein for biochemical characterization. Enzyme kinetics of mutant IDH protein correlated well with D-2-hydroxyglutarate production in cells with R132H exhibiting the highest and R132L the lowest K_M_ for α-ketoglutarate. Addition of D-2-hydroxyglutarate to the medium of cell lines revealed an inhibitory effect at higher concentrations. Migration of LN229 increased at lower D-2-hydroxyglutarate concentrations while higher concentrations showed no effect.

**Conclusion:**

These findings may suggest natural selection against the rare IDH1R132 mutations in human glioma due to toxicity caused by high levels of D-2-hydroxyglutarate.

## Background

Mutations in isocitrate dehydrogenases 1 or 2 (IDH1, IDH2) occur approximately in 75% of diffuse astrocytomas and oligodendroglial tumors [1-3], in 20% of acute myeloid leukemia (AML) [4,5], 50% of chondrosarcoma [6,7], 20% of intrahepatic cholangiocarcinoma [8] and in 20% of angioimmunoblastic T-cell lymphoma [9]. Besides overall frequency, also the type of IDH mutations differs in these tumor entities. In astrocytoma and oligodendroglioma more than 90% of all IDH mutations are of the IDH1R132H type [2], with the second most frequent type (approximately 4% of mutations) being IDH1R132C. The remaining mutations split into other very rare IDH1 and IDH2 mutations. Quite differently, IDH2R140Q mutations are most frequent in AML [5,10]. In chondrosarcoma and intrahepatic cholangiocarcinoma, the R132C alteration in IDH1 is the most frequent mutation [6,8], while in angioimmunoblastic T-cell lymphoma mutations in IDH2 are predominant [9]. Common to all IDH mutations is a neo-enzymatic activity of the mutated proteins that results in reduction of α-ketoglutarate (α-KG) to D-2-hydroxyglutarate (2-HG) with consumption of NADPH. The neo-enzymatic activity of mutated IDH proteins is believed to constitute the major tumorigenic mechanism of this alteration due to the inhibitory effect of 2-HG on α-KG dependent dioxygenases. Among these, the TET hydroxylases are affected resulting in a severely altered methylation pattern of DNA [11,12] conferring an unknown advantage to these tumor cells.

In astrocytoma and oligodendroglioma, we detected higher 2-HG levels in rare IDH1 mutations than in the most common IDH1R132H alteration [13]. A similar observation was made for IDH2, with cells carrying mutations affecting R172 exhibiting higher 2-HG concentrations than those with mutations in R140 [14]. These findings suggest different enzyme activity grades for the variant IDH mutants.

To explore the potential role of different mutation types in the formation of astrocytomas and oligodendroglial tumors, we determined the enzyme kinetics of wild type IDH1 and with the mutations R132H, R132C, R132G, R132S, R132L and R100Q followed by quantification of 2-HG in transfected cells and in human tumor tissues, and examined the effects on viability.

## Methods

### FFPE samples, DNA extraction and IDH1 sequencing

The collection of IDH1 mutant tumors consist out of previously published specimen [1,2,15] and tumors collected at the neuropathology department of the pathological institute of Heidelberg.

The DNA extractions form FFPE samples were performed by hand using Invisorb Genomic DNA Kit II (Invitek, Berlin, Germany) or semi-automated using Maxwell® 16 FFPE Plus LEV DNA Purification Kit (Promega, Madison, USA) following the manufacturer’s protocol.

The sequencing of published specimen was performed as described in the earlier publications. Our most recent probes were sequenced by generating a fragment of 212 bp length spanning the catalytic domain of IDH1 including codon 100 and 132 using 60 ng each of the sense primer IDH1-JM_f TGATGAGAAGAGGGTTGAGGA and the antisense primer IDH1-JM_r GCAAAATCACATTATTGCCAAC. PCR using standard buffer conditions, 20 ng of DNA and GoTaq DNA Polymerase (Promega, Madison, USA) employed 35 cycles with denaturing at 95°C for 30 s, annealing at 57°C for 40 s and extension at 72°C for 50 s in a total volume of 15 μl.

A total of 2 μl of the PCR amplification product was submitted to the sequencing reaction using the BigDye Terminator v3.1 Sequencing Kit (Applied Biosystems, Foster City, USA). Twenty-five cycles were performed employing 12 ng of the sense primer IDH1-JM_f TGATGAGAAGAGGGTTGAGGA, with denaturing at 95°C for 30 s, annealing at 57°C for 15 s and extension at 60°C for 240 s. A second round of sequencing analysis was performed using the antisense primer IDH1-JM_r GCAAAATCACATTATTGCCAAC and the sequencing reaction conditions as described above. Sequences were determined using the semi-automated sequencer (ABI 3100 Genetic Analyzer, Applied Biosystems, Foster City) and the Sequence Pilot version 3.1 (JSI-Medisys, Kippenheim, Germany) software.

### IDH1 mutant generation and cloning

IDH1 mutants were generated using the site directed mutagenesis method. Therefore, primers for each mutation were created and used on IDH1 wt cDNA in pDONR221 (DKFZ clone repository). Each mutation was confirmed by Sanger sequencing using the same procedure as described for the FFPE samples. The pDONR221 clones were used for all further LR-reactions into the described destination vectors. LR-reactions were performed following the manufacturers protocol (Invitrogen, Carlsbad, USA).

### IDH1 protein purification

To purify the different IDH1 proteins, the cDNAs were transferred into pDEST15 (Invitrogen, Carlsbad, USA), an *E. coli* expression vector containing a N-terminal GST tag (Invitrogen, Carlsbad, USA). The pDEST15 vectors were then transfected into *E.coli* expression strain KRX (Promega, Madison, USA). The *E.coli* were then streaked out on lysogeny broth (LB)-plates containing Ampicillin (100 μg/ml, Sigma-Aldrich, St. Luis, USA) and incubated at 37°C overnight. Six clones from each construct were transferred into 6 ml liquid LB containing 100 μg/ml Ampicillin and grown over night at 37°C in an orbital shaker (220 rpm). From these overnight cultures we used aliquots to prepare two small cultures (10 ml) for induction experiments. We only induced one culture from each colony following the manufacturer’s protocol (KRX protocol, Promega, Madison, USA). Briefly, inoculation of 1:100 of overnight culture, two hours incubation at 37°C and 220 rpm in the orbital shaker followed by the induction with 0.1% L-Rhamnose (10% stock solution in water, AppliChem, Gatersleben, Germany) and four hours incubation at RT and 220 rpm. Subsequently both cultures of each clone were harvested by centrifugation and resuspendet in GST-Lysis buffer from the Pierce® GST Spin Purification Kit (Thermo Scientific, Rockford, USA). Then they went through three freeze-thaw cycles with liquid nitrogen and a 15 min sonification step, followed by a 15 min centrifugation at 5000 rpm on 4°C. The supernatant was used for a SDS-PAGE (Invitrogen, Carlsbad, USA). A coomassie stain of the gel showed us which colonies yield best results. We then used these clones according to the protocol described above to prepare 200 ml cultures. The supernatant of these cultures were then used for protein purification with the Pierce® GST Spin Purification Kit (Thermo Scientific, Rockford, USA), following the manufacturer’s protocol.

### IDH1 enzyme kinetics

The measurement of the enzyme kinetics was performed with an Omega FluoStar (BMG Labtech, Ortenberg, Germany) equipped with a pump system, which was used to start the reaction, by adding 4 μg protein to the reaction mixture. All measurements were performed in 96-well plates (BD Falcon, Franklin Lakes, USA) in a total volume of 100 μl at 37°C. The reaction mixture consisted of Tris–HCl pH 7.4 (50 mM), MgCl_2_ (2 mM), NaCl (10 mM), BSA (0.05%), NADP+/NAPDH (10 mM or concentration row), and isocitrate/α-KG (10 mM or concentration row). As a negative control we used the same mixture, but without isocitrate or α-KG, depending on the K_M_ to be obtained. After the start of the reaction, a one second double orbital shaking (500 rpm) was used to mix the reaction. Thereafter, every two minutes data was obtained from each well measuring the NADPH production or consumption (Ex. 340 +/- 10 nm, Em. 440 +/- 10 nm), depending on the reaction analyzed. All measurements were done in triplicate and at least 15 data points of each reaction were used for K_M_ calculation. The mean of three independent measurements and the standard deviation of these is plotted for each K_M_.

### D-2-hydroxyglutarate

The 2-HG detection was performed with an enzymatic assay developed in our lab. Probe preparations and measurements were performed as described in [16].

D-2-hydroxyglutarate was obtained from Sigma Aldrich (St. Luis, USA) as disodium salt (catalogue number H8378).

Octyl-D-2-hydroxyglutarate was synthesized following the method reported by Xu et al. [12] with slight modifications (detailed protocol for D-2-hydroxyglutarate synthesis, see Additional file 1 and Additional file 2: Figure S1).

### Cell lines

All cell lines were achieved from the ATCC and cultured under standard culture conditions (37°C, 5% CO_2_) in DMEM medium with 1% Penicillin and Streptomycin and 10% fetal calf serum (all obtained from Gibco® Invitrogen, Carlsbad, USA).

For the generation of IDH1R132H overexpressing cells, IDH1R132H in the destination vector pDEST26 (N-terminal 6x His Tag) was used. LN229 cells were transfected with IDH1R132H in pDEST26 vector by Fugene 6 (Promega, Madison, USA) followed by picking of single cell clones. Single cell clones were selected with 2 mg/ml Gentamycin (Invitrogen, Carlsbad, USA). Clones that survived selection were analyzed for their expression of IDH1R132H by western blot. Clones H3 and H114 were chosen for further analysis, due to their different and stable expression levels of IDH1R132H.

For all experiments with the inducible expression system we used normal cell culture medium, but exchanged normal FCS with 10% Tet system approved FBS (Clontech, Mountain View, USA). To generate an inducible cell line we used the pT-REx-DEST system (Invitrogen, Carlsbad, USA). As a first step we transfected the cell line LN319 with pcDNA6/TR. From this transfection we generated single cell clones and tested their reliability by introducing EGFP in pT-REx-DEST30. We chose the clone with no GFP expression in tetracycline free media and with the highest expression after induction with 1 μM Doxycycline (Sigma-Aldrich, St. Luis, USA). This clone 09 (K09) was then used for all further experiments.

For the experimental setup we used LN319 K09 transfected with IDH1 wild type (wt), the different IDH1 mutants, and GFP in pT-REx-DEST30. All cell lines were seeded as described under proliferation analysis. For each cell line two triplicates were seeded. One was induced with 1 μg/ml Doxycycline, the other one was treated with the comparable amount of solvent (DMSO, Sigma-Aldrich, St. Luis, USA).

To generate cell lines which express IDH1 wt and mutant proteins, we used cDNAs in pMXs-GW-IRES-BsdR and transfected them into HEK293T cells with FuGene®. Cells were subsequently put under selection pressure, by adding 4 μg/ml Blasticidine S (Sigma-Aldrich, St. Luis, USA). As control we used GFP in pMXs-GW-IRES-BsdR.

### Cell line analysis

For cell number analysis CellTiterGlo (Promega, Madison, USA) was used in 96-well plate format. All cells were seeded at a density of 5,000 cells/well and subsequently treated. Measurements were performed at the indicated time points following the manufacturer’s protocol.

All migration assays were performed with Ibidis Culture-Inserts. In each well of the insert, 50,000 cells were seeded. After 24 h, the cells were treated with 10 μg/ml Mitomycin C (Sigma-Aldrich, St. Luis, USA) for 2 h, to avoid the influence of proliferation in the assay. Afterward, the inserts were removed leaving a gap of 500 μm +/- 50 μm. Cells were washed with PBS once and thereafter normal medium was applied. This medium was subsequently substituted with 2-HG at the given concentrations. The gap was microscopically documented at the start and the indicated time points. The pictures were analyzed with TScratch (http://chaton.ethz.ch/software/) and the area closed after the indicated time point was plotted in the graph.

The soft agar assays were performed in 6-well plates. The wells were prepared with a 1% agar solution (Agar noble, US Biological, Salem, USA) as base agar. On top of this, 5,000 cells in 0.35% agar were seeded and grown for 14 days. The cells were stained with 0.00025% crystal violet solution and colonies > 1 mm^2^ were counted.

## Results

### Distribution of IDH1 mutations in gliomas

**Figure 1:**
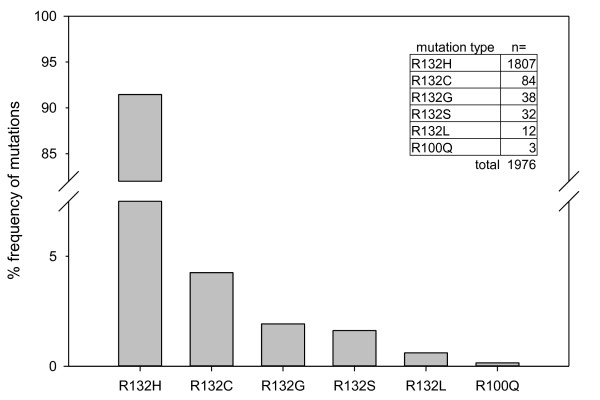
**Distribution of IDH1 mutation types.** Distribution of mutation types in the Heidelberg series of 1,976 diffuse astrocytomas and oligodendrogliomas with *IDH1* mutations.

Including data from a previous study [2] and on ongoing routine analysis of all diffuse astrocytoma and oligodendroglioma in the Department of Neuropathology at the University Heidelberg, the present series of IDH1 mutated gliomas comprises tumors from 1,976 patients. These 1,976 tumors represent only the immunohistochemical and sequence verified IDH1 mutant cases out of the whole collection of glial tumors in our department. The frequency of IDH1 mutations in these tumors decreases in the order R132H (91.5%), R132C (4.3%), R132G (1.9%), R132S (1.6%), R132L (0.6%) and R100Q (0.3%). Incidences of the mutations are given in Figure 1.

### 2-HG in tumor tissue

**Figure 2:**
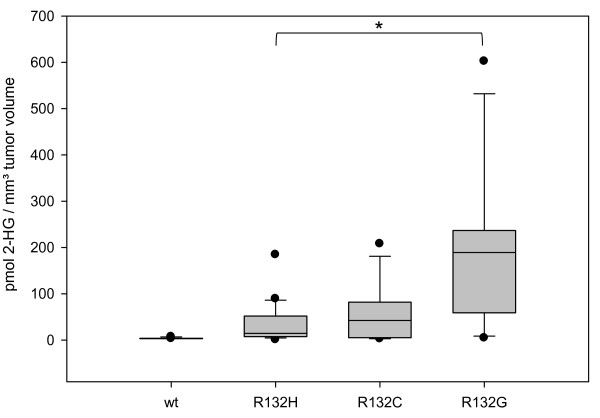
**D-2-hydroxyglutarate levels in FFPE tissue. **D-2-hydroxyglutarate levels in FFPE tissue from diffuse astrocytomas and oligodendrogliomas with *IDH1* mutations. Shown are the levels of 6 IDH1 wt tumors, 25 with IDH1R132H, 8 with IDH1R132C and 7 with IDH1R132G. All mutant tumors show significant (** = p ≤ 0.01) higher 2-HG levels than wildtype tumors, but only the difference between R132H and R132G mutation was statistically significant (* = p ≤ 0.05).

We determined the amount of 2-HG in glioma tissue. The limiting step of our analyses was the low incidence of IDH1 mutations in gliomas other than R132H. Sufficient formalin fixed and paraffin embedded tissue was available from 9 tumors with R132C, 6 tumors with R132G and 1 each with R132S and R132L. Due to loss of 2-HG during the processing up to paraffin embedding [13], only approximate values for 2-HG could be determined. Lowest 2-HG levels were detected in tumors with R132H followed by R132C and then R132G. The single tumors with R132S and R132L mutation also exhibited high levels of 2-HG. No 2-HG was detected in 5 control glioma samples without IDH1 mutation. The data is shown in Figure 2.

### Enzyme kinetics

**Figure 3:**
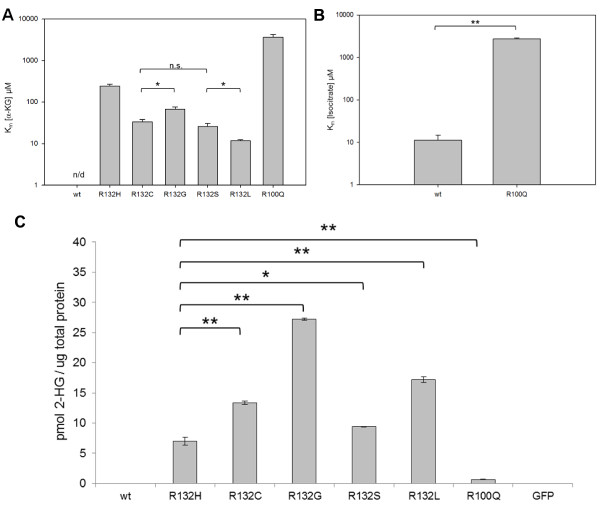
**K**_**M **_**values and D-2-hydroxyglutarate levels of IDH1 mutation types.** K_M_ values of IDH1 mutation types for α-ketoglutarate **(A)** and isocitrate **(B)**. The K_M_ value for α-ketoglutarate of IDH1 wt and the K_M_ values for isocitrate of all IDH1^R132^ mutations could not be determined. Therefore K_M_ values of R132 mutations are not shown. All K_M_ values are significantly different from each other with a p-value of ≤0.01, if not otherwise stated (* = p ≤ 0.05 or n.s. = not significant). All measurements were done in triplicate from different protein purifications. D-2-hydroxyglutarate levels in HEK293T cells ectopically expressing different IDH1 mutations and wild type IDH1 **(C)**. Levels are measured in cells from one confluent dish of 6-well Plate 72 h after seeding. All D-2-hydroxyglutarate levels of the mutant IDH1 expressing cells are significantly higher (p ≤ 0.01) than both controls (wt and GFP). The levels of all other R132 mutants are significantly higher than the one of R132H (p-value: * = p ≤ 0.05 and ** = p ≤ 0.01). Only the level of R100Q is significant lower than the level of R132H (p-value: ** = p ≤ 0.01).

Recombinant expression of proteins with IDH1 mutations followed by purification enabled the determination of Michaelis constants for each mutant protein with the substrate α-KG. A high K_M_ of α-KG was observed for the R132H mutation (243.7 μM) whereas lower K_M_’s were seen for R132G (67.4 μM), R132C (33.7 μM), R132S (26.1 μM) and R132L (11.8 μM). Data is shown in Figure 3A.

The very rare R100Q mutation exhibited a high K_M_ of (3671.1 μM). This was the only mutated IDH1 protein for which a Michaelis constant for isocitrate was detected (K_M_ = 2724.0 μM) (Figures 3A and 3B). To further validate this data, we transfected HEK293T cells with all different IDH1 variants and determined the cellular 2-HG level (Figure 3C). As expected the R132C mutant produced more 2-HG than R132H mutant. Also, the other mutants R132G, R132S and R132L all produced more than R132H. However, the 2-HG concentration measured did not exactly correspond to the K_M_ values determined. The 2-HG concentration from R100Q was as low as expected, ranging below that from R132H. To exclude the possibility that the different 2-HG concentrations are due to differential expression of the respective mutant proteins or different protein stabilities, we performed a western blot demonstrating comparable protein quantity (Additional file 3: Figure S5).

### Cell viability dependence on 2-HG concentration

**Figure 4:**
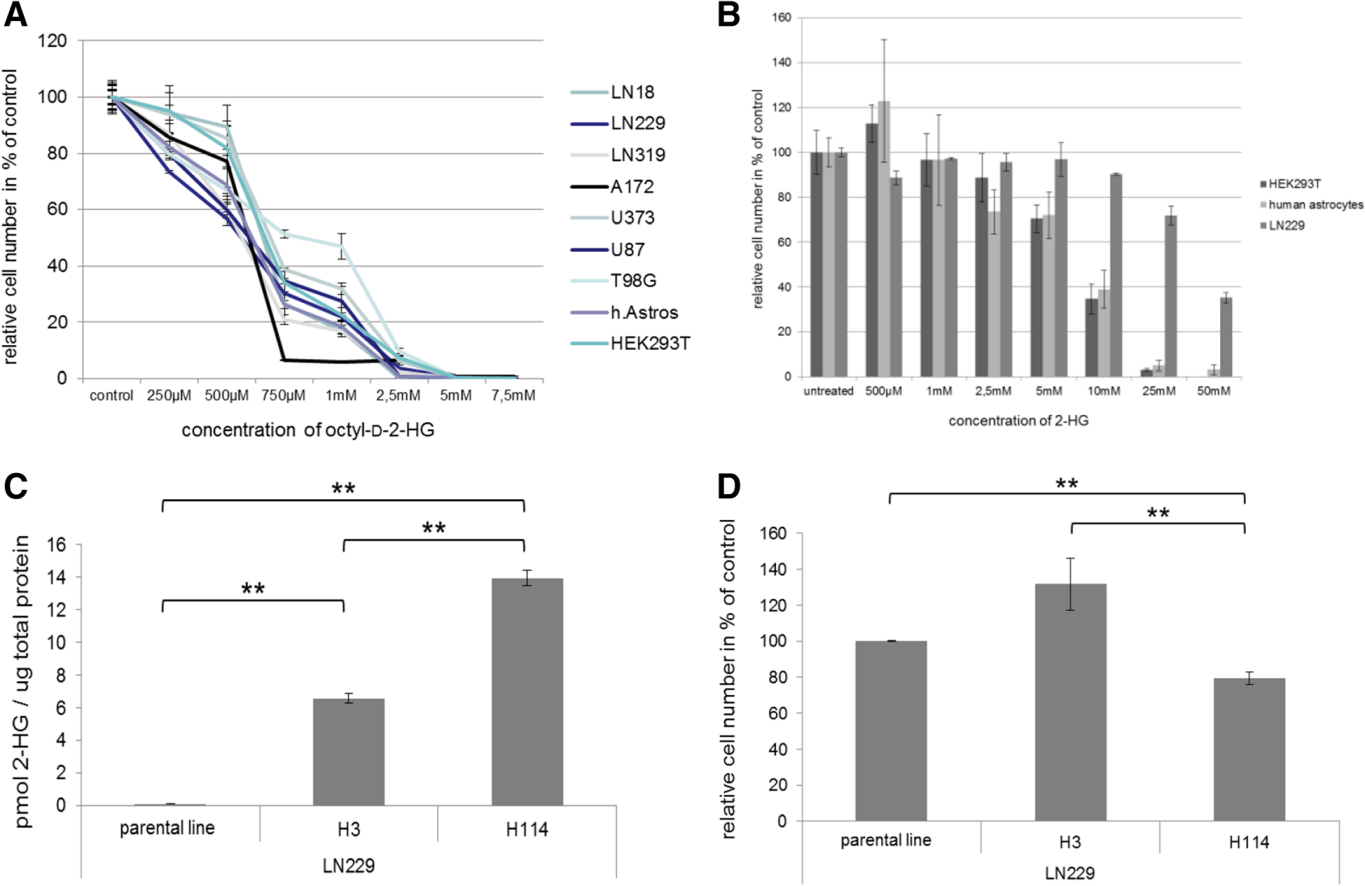
**D-2-hydroxyglutarate toxicity and analysis of two LN229 sublines.** Octyl-D-2-hydroxyglutarate mediated toxicity in different cell lines **(A)**. D-2-hydroxyglutarate mediated toxicity in different cell lines **(B)**. Toxicity was measured 48 h after treatment. Plotted are the relative cell numbers in % compared to the corresponding untreated control. D-2-hydroxyglutarate levels in LN229 glioma cell line and two LN229 glioma cell subclones H3 and H114 overexpressing different protein levels of R132H **(C)**. All D-2-hydroxyglutarate levels are significantly different from each other (p ≤ 0.01). Proliferation of LN229 glioma cell line and of two LN229 glioma cell subclones H3 and H114 overexpressing different protein levels of R132H **(D)**. The relative cell numbers were determined 48 h after seeding and are plotted in % compared to the corresponding control. The enhanced proliferation of subclone H3 is not significant compared to the parental line, but the reduced proliferation of subclone H114 is statistically significant compared to both other lines (p ≤ 0.01).

Viability upon exogenous exposure to 2-HG was tested using immortalized human astrocytes, HEK293T cells, and seven glioma lines (LN18, LN229, LN319, A172, U87, U373, T98G) by addition of cell permeable octyl-2-HG to medium [12]. To determine the concentration range to be used, we calculated the molar 2-HG concentrations in the tumor specimens from previous reports [17,18] and our tumor material. For converting concentrations given in μmol/g to molarities we assumed brain tumor tissue density to be similar to normal human brain tissue measuring 1.054 +/- 0.014 g/cm^3^[19]. On this basis, 2-HG concentrations in brain tumor tissues range from 0.1–126 mM. We, therefore, added cell permeable octyl-2-HG to cell medium in concentrations ranging from 100 μM to 150 mM. All cell lines challenged perished at 2-HG concentrations of 7.5 mM and above. Therefore, only data using octyl-2-HG concentrations ranging from 250 μM to 7.5 mM are shown in Figure 4A.

Due to the high toxicity of octyl-2-HG we switched to the less cell permeable sodium salt of 2-HG, because we aimed for more physiological concentrations in our experiments. We added this 2-HG to HEK293T, human astrocytes and LN229 in concentrations ranging from 500 μM to 50 mM. Concentrations at the low end from 500 μM to 1 mM had no significant effect on proliferation. From 2.5 mM to 50 mM, increasing toxicity was observed with LN229 being more resistant than HEK293T and human astrocytes (Figure 4B).

In order to test the effect of endogenous 2-HG we transfected LN229 with pDEST26 containing an IDH1R132H construct. Two clones H3 and H114 were established by single cell cloning, both stably producing 2-HG, albeit at different levels. Clone LN229 H3 contained 6.58 pmol 2-HG/μg total protein, a concentration not significantly affecting proliferation. In fact, a trend for higher proliferation was noted. In contrast, clone LN229 H114 containing 13.94 pmol 2-HG/μg total protein exhibited a significant reduction of proliferation (Figures 4D).

### Concentration dependent toxicity of different IDH1 mutations

We transfected in LN319 and HEK293T cells with pcDNA6/TR and selected single colonies. This was done to have cell lines with high expression of TR (tet repressor) sufficient for silencing pT-Rex-DEST30 prior to induction with Doxycycline.

We cloned IDH1 wt, all the R132 mutants and the R100Q mutant in pT-Rex-DEST30, and transfected these constructs into LN319 and HEK293T containing pcDNA6/TR. Then, the expression of the respective mutant IDH protein was induced.

**Figure 5:**
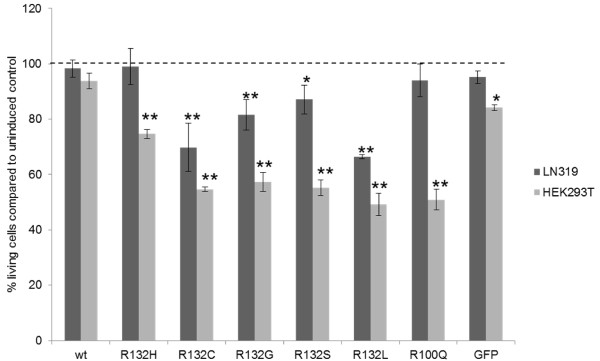
**Proliferation analysis of LN319 glioma and HEK293T cells with and without induction of mutant IDH expression.** Cell viability of LN319 glioma and HEK293T cells bearing tetracycline inducible constructs of different IDH mutations and controls 72 h after induction. Shown are the relative cell numbers compared to the corresponding uninduced controls. The significant differences are depicted in the figure with their corresponding to their p-values (* = p ≤ 0.05 and ** = p ≤ 0.01).

Induction of R132H had no effect on proliferation in LN319, but in HEK293T it lead to a reduction in growth. Induction of all the mutations with lower K_M_ for α-KG resulted in reduced cell growth in both lines. As expected, expression of the R100Q mutation, producing low levels of 2-HG, also had no effect on growth in LN319. Neither expression of wt IDH1 protein nor of GFP affected cell growth in LN319. Results of growth kinetics for the different transfectants are shown in Figure 5.

### Effect of 2-HG concentration on migration and colony formation

**Figure 6:**
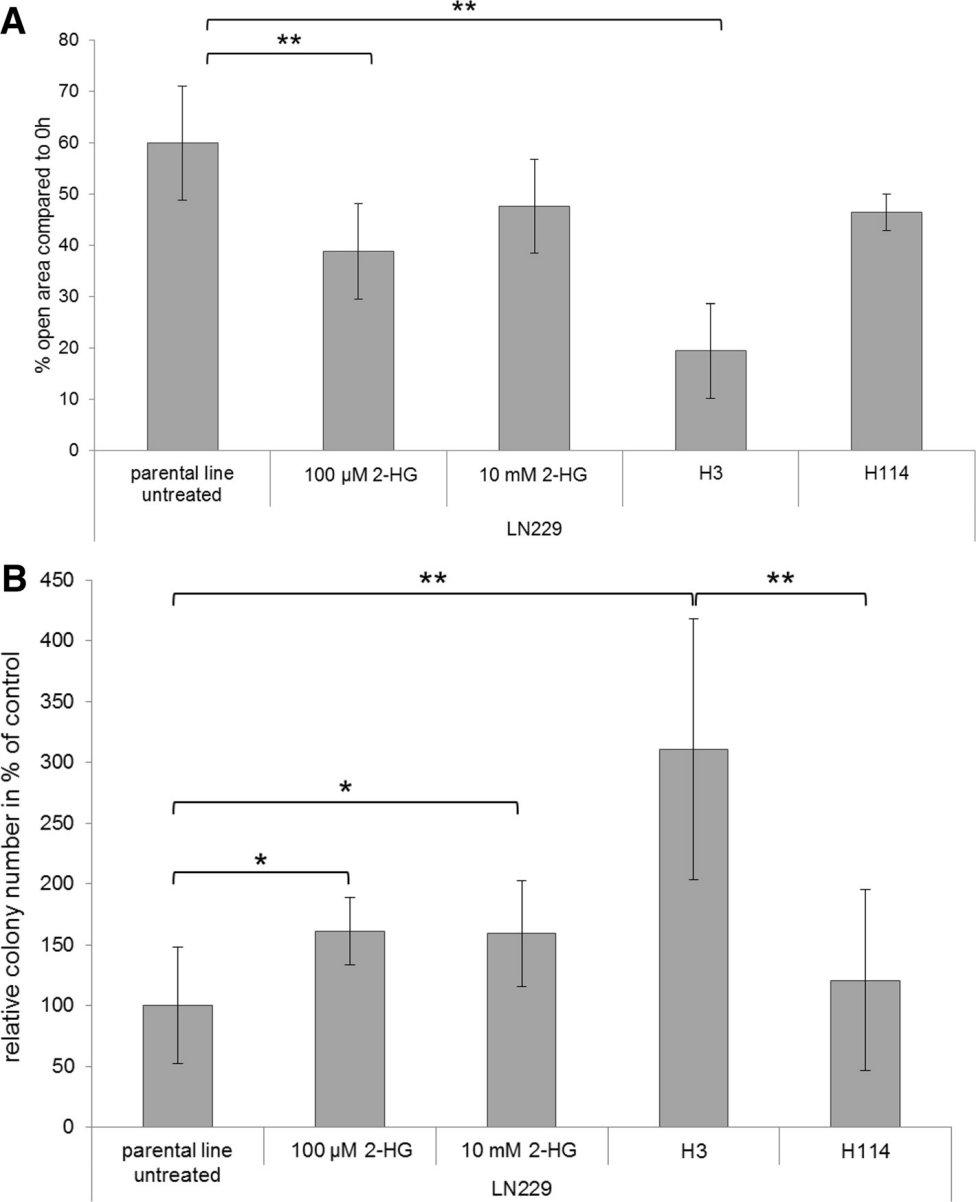
**Migration and soft agar analysis of LN229 treated with D-2-hydroxyglutarate and LN229 subclones.** Migration of LN229 and subclones H3 and H114 after 72 h. LN229 cells were treated with 100 μM or 10 mM 2-HG. Subclones H3 and H114 expressed R132H protein in different amounts **(A)**. Plotted is the open area after 24 h of incubation. Treatment with 100 μM 2-HG or ectopic expression of low amounts of R132H protein (subclone H3) increased migration speed significantly (** = p ≤ 0.01). Colony formation in soft agar of the same cells in the same setting **(B)**. Shown are the relative colony numbers compared to the untreated parental line (100%). The 2-HG treatment could significantly (* = p ≤ 0.05) increase colony formation independent of the used concentration. An even stronger increase (** = p ≤ 0.01) could be measured for subclone H3, but no difference could be detected for subclone H114.

Wound healing assays were performed employing LN229 and different concentrations of 2-HG. In addition, we performed this assay employing LN229 H3 and LN229 H114 cells with ectopic expression of mutant R132H IDH protein at different levels. Addition of 2-HG at low concentration of 100 μM resulted in enhanced migration, which was less pronounced at higher concentrations of 10 mM. LN229 H3 showed a migration rate higher than the parental LN229 while LN229 H114 did not differ from LN229 in this respect (Figure 6A).

Colony formation in soft agar was augmented upon exposure to 2-HG in moderate concentrations. Concentrations of 100 μM and 10 mM 2-HG in medium both resulted in an increase of colonies by 61% and 59%, respectively. LN229 H3 containing 6.58 pmol 2-HG/μg total protein exhibited a 3-fold increase in colony formation. In contrast, LN229 H114 containing 13.94 pmol 2-HG/μg total protein did not form significantly more colonies than LN229 without treatment (Figure 6B).

## Discussion

The IDH1 mutation of the R132H type by far outnumbers the other IDH1 mutations in diffuse astrocytic and oligodendroglial tumors. In order to provide a hypothesis for this lopsided distribution, we characterized biochemical features of different IDH1 mutations, analyzed native tumors and performed experiments addressing the effects of different mutations on *in vitro* systems.

Common to all IDH1 mutations is acquisition of a neo-enzymatic activity with the ability to convert α-KG to 2-HG. Thus, we determined the Michaelis constants of mutated IDH proteins for the substrate α-KG. We detected the highest K_M_ for α-KG in protein carrying the R132H mutation followed by R132G, R132C, R132S and R132L (Figure 3). While increased 2-HG levels are expected to provide a selection advantage for early tumor cells, it should be kept in mind that high 2-HG levels may be toxic. Thus, positive discrimination for a mutation type resulting in a protein with moderate activity for features beneficial at low but potentially deleterious at high dose is well compatible with a selection process.

A beneficial effect of moderately increased 2-HG on proliferation (cell line LN229 H3, 6.58 pmol 2-HG/μg total protein) is demonstrated in Figure 4D. In contrast, strongly increased 2-HG levels (cell line LN229 H114, 13.94 pmol 2-HG/μg total protein) did not favor proliferation. Similarly, migration could be increased for LN229 by 100 μM 2-HG in the medium and slightly less by 10 mM 2-HG in the medium. Moreover, the cell line LN229 H3 with moderately induced 2-HG production exhibited increased migration compared to LN229. LN229 H114, with higher 2-HG production, did not migrate significantly faster than LN229 (Figure 6A). Likewise, colony formation was strongly supported by moderate 2-HG concentrations in the LN229 and subclone LN229 H3. In contrast, LN229 H114, producing high levels of 2-HG, exhibited no benefit (Figure 6B).

To test the effect of 2-HG on viability of cells we added 2-HG, or the more cell permeable octyl-2-HG [12], to culture medium. In our models employing human astrocytes and HEK293T, LD_50_ for 2-HG was determined for concentrations between 5 mM and 10 mM while the LD_50_ for LN229 was approximately 50 mM (Figure 4B). In the same cell lines and several more, the LD_50_ for octyl-2-HG was reached at approximately 750 μM (Figure 4A). Moreover, transfection and induction of the different IDH1 mutations into LN319 cells demonstrated no influence on proliferation for R132H; however, the other mutations with lower K_M_ for α-KG inhibited proliferation. These findings demonstrated considerable cell toxicity of 2-HG.

To further support this hypothesis we analyzed the amount of 2-HG in formalin fixed and paraffin embedded brain tumors harboring different IDH mutations. The limiting step of our analyses was the low incidence of IDH1 mutations other than R132H in gliomas. The detection of moderately increased 2-HG levels in tumors with the IDH1R132H mutation and higher levels in the rare mutation types matched well with the different affinities for α-KG of the respective mutations.

Thus, in glioma cells we demonstrate beneficial effects of moderate 2-HG concentrations for proliferation, migration and colony formation and toxic effects on these readouts for high 2-HG concentrations. This would well match a model favoring an IDH1 mutation with an intermediate K_M_ for α-KG.

These findings may explain the strong preponderance of the IDH1R132H mutation type in glioma.

Both exo- and endogenous mechanisms affect types of mutation in DNA. Sporadic primary brain tumors in humans have not been shown to be associated with typical exogenous DNA damage such as ultraviolet-induced DNA-damage or that following exposure to carcinogens. It is unresolved whether accumulation of distinct mutations such as IDH1 originates from an endogenously mediated increase or a specific failure of repairing this distinct alteration. In low grade glioma, cytosine to thymidine transitions constitute the most frequent mutation type on the single nucleotide level [20]. R132H facilitated by a CGT to CAT change corresponds to a C to T transition on the reverse strand. On the other hand, R132C facilitated by CGT to TGT is based on this transition on the coding strand. Neglecting potential repair of the C to T transition on the reverse strand during replication, R132H and R132C could be expected to occur with comparable frequency. This holds true for these mutations in acute myeloid leukemia [5,21]. In contrast, IDH1R132H dominates in diffuse gliomas and R132C appears to be the most frequent mutation in chondrosarcoma [6,7] and intrahepatic cholangiocarcinoma [8,22]. Thus, the strong bias for IDH1R132H mutation in astrocytoma and oligodendroglioma may support a selection bias for this alteration.

## Conclusion

We determined significantly different enzymatic activities for distinct IDH1 mutations and provide a selection based hypothesis for the preponderance of the IDH1R132H mutation in astrocytoma and oligodendroglioma.

## Competing interests

AvD, JB and SP hold a patent for the enzymatic D-2-hydroxyglutarate detection assay. All other authors declare that they have no competing interests.

## Authors’ contributions

Conceived and designed the experiments SP. Generated mutant proteins and vectors SP, inducible cell line JL SP, over expression cell lines SW AB SP. Performed soft agar assay and migration assay LS, proliferation analysis SP, enzyme kinetics AB JL JB, 2-HG detection assay AB JB, octyl-2-HG synthesis AM, database analysis AvD, statistical analysis LS SP. Wrote the manuscript AvD LS SP. All authors read and approved the final manuscript.

## Supplementary Material

Additional file 1 **d-2-hydroxyglutarate synthesis.** Click here for file

Additional file 2: Figure S1 Synthetic route to octyl-D-2-HG. Click here for file

Additional file 3: Figure S5 Western blot analysis of overexpression cell lines. Click here for file

## References

[B1] Balss J Meyer J Mueller W Korshunov A Hartmann C von Deimling A Analysis of the IDH1 codon 132 mutation in brain tumors Acta Neuropathol 2008 2 597 602 10.1007/s00401-008-0455-2 18985363

[B2] Hartmann C Meyer J Balss J Type and frequency of IDH1 and IDH2 mutations are related to astrocytic and oligodendroglial differentiation and age: a study of 1010 diffuse gliomas Acta Neuropathol 2009 2 469 474 10.1007/s00401-009-0561-9 19554337

[B3] Yan H Parsons DW Jin G IDH1 and IDH2 mutations in gliomas N Engl J Med 2009 2 765 773 10.1056/NEJMoa0808710 19228619 PMC2820383

[B4] Mardis ER Ding L Dooling DJ Recurring mutations found by sequencing an acute myeloid leukemia genome N Engl J Med 2009 2 1058 1066 10.1056/NEJMoa0903840 19657110 PMC3201812

[B5] Paschka P Schlenk RF Gaidzik VI IDH1 and IDH2 mutations are frequent genetic alterations in acute myeloid leukemia and confer adverse prognosis in cytogenetically normal acute myeloid leukemia with NPM1 mutation without FLT3 internal tandem duplication J Clin Oncol 2010 2 3636 3643 10.1200/JCO.2010.28.3762 20567020

[B6] Amary MF Bacsi K Maggiani F IDH1 and IDH2 mutations are frequent events in central chondrosarcoma and central and periosteal chondromas but not in other mesenchymal tumours J Pathol 2011 2 334 343 10.1002/path.2913 21598255

[B7] Arai M Nobusawa S Ikota H Takemura S Nakazato Y Frequent IDH1/2 mutations in intracranial chondrosarcoma: a possible diagnostic clue for its differentiation from chordoma Brain Tumor Pathol 2012 2 201 206 10.1007/s10014-012-0085-1 22323113

[B8] Borger D Tanabe K Fan K Frequent mutation of isocitrate dehydrogenase (IDH)1 and IDH2 in cholangiocarcinoma identified through broad-based tumor genotyping Oncol 2012 2 72 79 10.1634/theoncologist.2011-0386 PMC3267826 22180306

[B9] Cairns RA Iqbal J Lemonnier F IDH2 mutations are frequent in angioimmunoblastic T-cell lymphoma Blood 2012 2 1901 1903 10.1182/blood-2011-11-391748 22215888 PMC3293643

[B10] Ward PS Patel J Wise DR The common feature of leukemia-associated IDH1 and IDH2 mutations is a neomorphic enzyme activity converting alpha-ketoglutarate to 2-hydroxyglutarate Cancer Cell 2010 2 225 234 10.1016/j.ccr.2010.01.020 20171147 PMC2849316

[B11] Turcan S Rohle D Goenka A IDH1 mutation is sufficient to establish the glioma hypermethylator phenotype Nature 2012 2 479 483 10.1038/nature10866 22343889 PMC3351699

[B12] Xu W Yang H Liu Y Oncometabolite 2-hydroxyglutarate is a competitive inhibitor of alpha-ketoglutarate-dependent dioxygenases Cancer Cell 2011 2 17 30 10.1016/j.ccr.2010.12.014 21251613 PMC3229304

[B13] Sahm F Capper D Pusch S Balss J Koch A Langhans C Okun J von Deimling A Detection of 2-Hydroxyglutarate in formalin-fixed paraffin-embedded glioma specimens by gas-chromatography/mass-spectrometry Brain Pathol 2012 2 26 31 10.1111/j.1750-3639.2011.00506.x 21631627 PMC8028858

[B14] Ward PS Lu C Cross JR Abdel-Wahab O Levine RL Schwartz GK Thompson CB The potential for isocitrate dehydrogenase mutations to produce 2-hydroxyglutarate depends on allele specificity and subcellular compartmentalization J Biol Chem 2013 2 3804 3815 10.1074/jbc.M112.435495 23264629 PMC3567635

[B15] Capper D Weissert S Balss J Characterization of R132H Mutation Specific IDH1 Antibody binding in brain tumors Brain Pathol 2010 2 245 254 10.1111/j.1750-3639.2009.00352.x 19903171 PMC8094636

[B16] Balss J Pusch S Beck A-C Enzymatic assay for quantitative analysis of (D)-2-hydroxyglutarate Acta Neuropathol 2012 2 883 891 10.1007/s00401-012-1060-y 23117877

[B17] Dang L White DW Gross S Cancer-associated IDH1 mutations produce 2-hydroxyglutarate Nature 2009 2 739 744 10.1038/nature08617 19935646 PMC2818760

[B18] Juratli TA Peitzsch M Geiger K Schackert G Eisenhofer G Krex D Accumulation of 2-hydroxyglutarate is not a biomarker for malignant progression in IDH-mutated low-grade gliomas Neuro Oncol 2013 2 682 690 10.1093/neuonc/not006 23410661 PMC3661092

[B19] Barber TW Brockway JA Higgins LS The density of tissues in and about the head Acta neurologica Scandinavica 1970 2 85 92 10.1111/j.1600-0404.1970.tb05606.x 4983875

[B20] Lawrence MS Stojanov P Polak P Mutational heterogeneity in cancer and the search for new cancer-associated genes Nature 2013 2 214 218 10.1038/nature12213 23770567 PMC3919509

[B21] Gross S Cairns RA Minden MD Cancer-associated metabolite 2-hydroxyglutarate accumulates in acute myelogenous leukemia with isocitrate dehydrogenase 1 and 2 mutations J Exp Med 2010 2 339 344 10.1084/jem.20092506 20142433 PMC2822606

[B22] Kipp BR Voss JS Kerr SE Isocitrate dehydrogenase 1 and 2 mutations in cholangiocarcinoma Hum Pathol 2012 2 1552 1558 10.1016/j.humpath.2011.12.007 22503487

