# Supplementary material for: D-2-Hydroxyglutarate producing neo-enzymatic activity inversely correlates with frequency of the type of isocitrate dehydrogenase 1 mutations found in glioma
Source: Acta Neuropathol Commun. 2014 Feb 14;2:19. doi: 10.1186/2051-5960-2-19 (PMC3937031; doi:10.1186/2051-5960-2-19)

D-2-hydroxyglutarate synthesis

To a suspension of (R)-2-amino-5-(benzyloxy)-5-oxopentanoic acid (1.430 g, 6.027 mmol, Merck) in H_2O (48 mL) and AcOH (12 mL) was added a freshly prepared aqueous solution of $NaNO_2$ (12 mL, 1.0 M, 12 mmol) dropwise at 0°C with stirring. After the addition was complete, the reaction mixture was allowed to warm to RT, wherein the reaction mixture clarified. After stirring for 18 h, the solvent was removed on a rotary evaporator (50 mBar, 48°C water bath) and the resulting solid was dried under high vacuum to give (R)-5-(benzyloxy)-2-hydroxy-5-oxopentanoic acid as a white solid. To a solution of this dried crude product in anhydrous DMF (12 mL) was added $NaHCO_3$ (507 mg, 6.03 mmol) followed by octyl iodide (2.20 mL, 12.1 mmol, Sigma) at RT under argon. The reaction vessel was wrapped in foil and stirred in the dark. After 18 h, the reaction mixture was diluted with H_2O (200 mL) and EtOAc (50 mL). The two layers were separated and the aqueous layer was extracted with EtOAc (3 x 50 mL). The combined organic layers were dried ($MgSO_4$), filtered, concentrated, and purified by column chromatography (0–30% gradient of EtOAc in petroleum ether) to give 631 mg (60%, 2 steps) of (R)-5-benzyl 1-octyl 2-hydroxypentanedioate as a colorless oil:

R_f 0.34 (20% EtOAc in petroleum ether)

^{1}H NMR (400 MHz, $CDCl_3$): δ 7.39–7.29 (m, 5 H), 5.13 (s, 2 H), 4.22 (ddd, *J* = 8.0, 5.4, 4.2 Hz, 1 H), 4.17 (t, *J* = 6.8 Hz, 2 H), 2.84 (d, *J* = 5.4 Hz, 1 H), 2.57 (ddd, *J* = 16.5, 8.4, 7.0 Hz, 1 H), 2.49 (ddd, *J* = 16.5, 8.4, 6.0 Hz, 1 H), 2.20 (dddd, *J* = 14.1, 8.5, 7.1, 4.2 Hz, 1 H), 1.95 (dddd, *J* = 14.2, 8.2, 8.2, 6.1 Hz, 1 H), 1.70–1.61 (m, 2 H), 1.38–1.21 (m, 10 H), 0.88 (t, *J* = 6.8 Hz, 3 H) ppm (supplement figure 2).

To a solution of (R)-5-benzyl 1-octyl 2-hydroxypentanedioate (1.259 g, 3.593 mmol) in EtOAc (28 mL, 0.13 M) was added 10% Pd/C (150 mg) under argon. The atmosphere was replaced with H_2 (balloon pressure) and, after 18 h, the reaction mixture was filtered through celite, washing the celite with EtOAc (3 x 30 mL). The resulting clear and colorless filtrate was concentrated and the product was purified by column chromatography (0–100% gradient of EtOAc in petroleum ether) to give 875 mg (94%) of octyl-D-2-hydroxyglutarate as a white solid:

R_f 0.30 (3% MeOH, 0.5% AcOH in CH_2Cl_2)

mp 36.8–37.4°C

^{1}H NMR (600 MHz, $CDCl_3$): δ 4.24 (dd, *J* = 8.0, 4.2 Hz, 1 H), 4.19 (t, *J* = 6.8 Hz, 2 H), 2.93 (bs, 1 H), 2.56 (ddd, *J* = 16.8, 8.4, 7.0 Hz, 1 H), 2.49 (ddd, *J* = 16.8, 8.4, 6.0 Hz, 1 H), 2.19 (dddd, *J* = 14.1, 8.3, 7.1, 4.2 Hz, 1 H), 1.94 (dddd, *J* = 14.1, 8.1, 8.1, 6.1 Hz, 1 H), 1.71–1.62 (m, 2 H), 1.40–1.21 (m, 10 H), 0.88 (t, *J* = 6.8 Hz, 3 H) ppm (supplement figure 3).

^{13}C NMR (125 MHz, $CDCl_3$): δ 178.8, 174.9, 69.5, 66.3, 31.9, 29.5, 29.3 (2 overlapping signals), 29.2, 28.6, 25.9, 22.7, 14.2 ppm (supplement figure 4).

HRMS–TOF ES (*m/z*): $[M + H]^+$ calcd for $C_{13}H_{25}O_5$, 261.1702; found, 261.1714.

Supplement figure 2

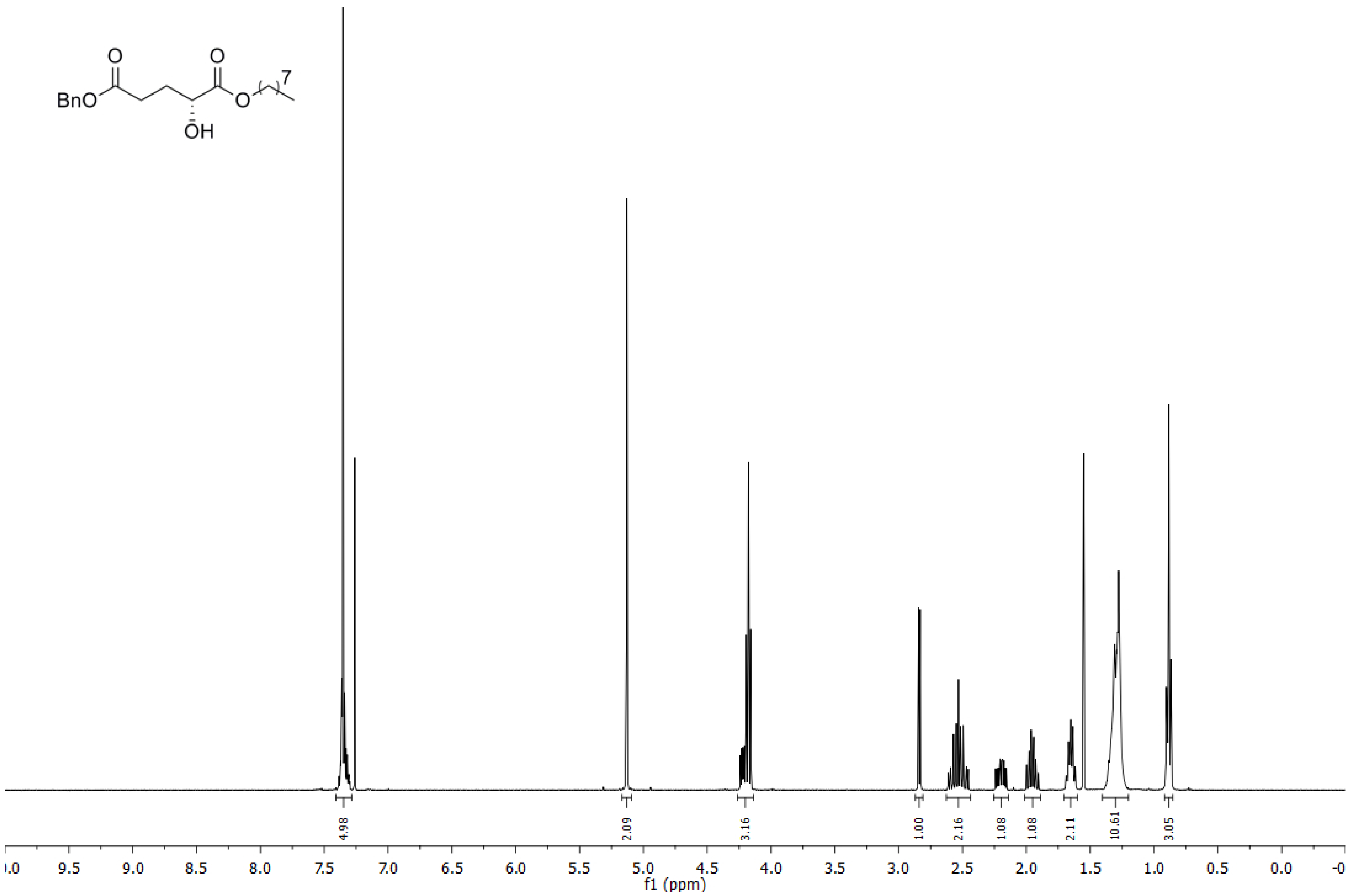

Supplement figure 3

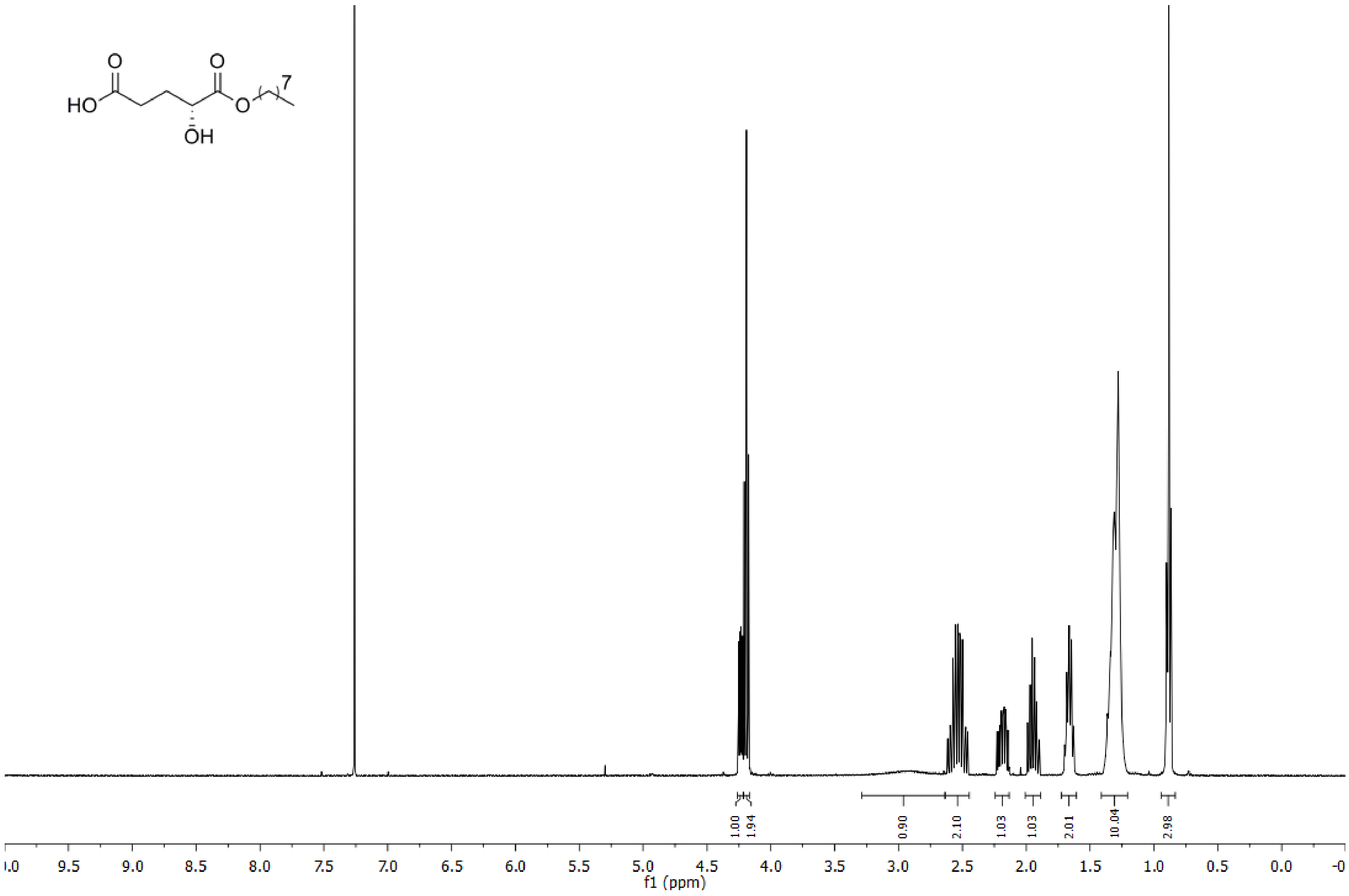

Supplement figure 4

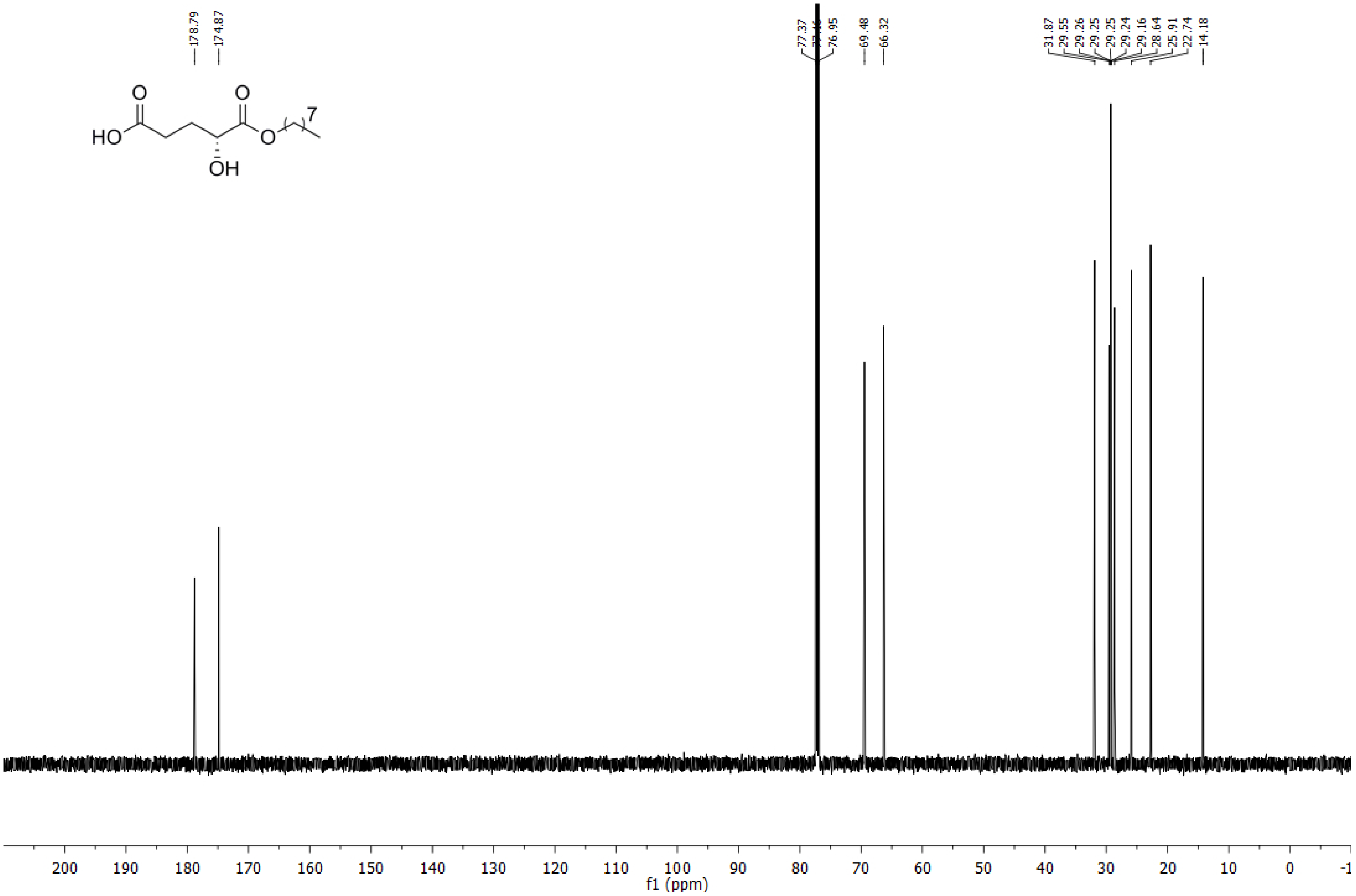

Supplement: Additional file 1 — d-2-hydroxyglutarate synthesis. [file 2051-5960-2-19-S1.pdf]
