# Supplementary figures and images for: D-2-Hydroxyglutarate producing neo-enzymatic activity inversely correlates with frequency of the type of isocitrate dehydrogenase 1 mutations found in glioma
Source: Acta Neuropathol Commun. 2014 Feb 14;2:19. doi: 10.1186/2051-5960-2-19 (PMC3937031; doi:10.1186/2051-5960-2-19)

# Supplement figure 1

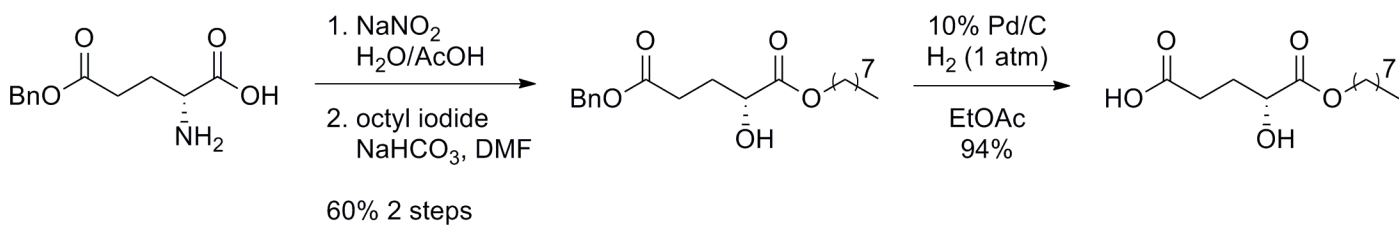

Supplement: Additional file 2: Figure S1 — Synthetic route to octyl-D-2-HG. [file 2051-5960-2-19-S2.pdf]

# Supplement figure 5

## HEK 293T

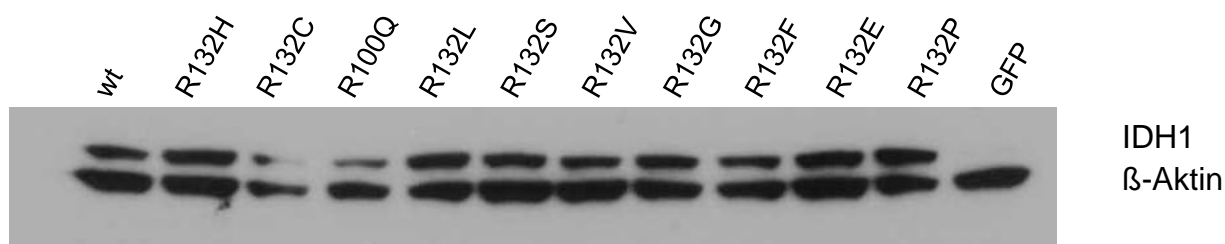

Supplement: Additional file 3: Figure S5 — Western blot analysis of overexpression cell lines. [file 2051-5960-2-19-S3.pdf]
